# Outpatient dental procedures carried out by Pediatric Dentists within Brazil’s public healthcare system

**DOI:** 10.1590/1807-3107bor-2024.vol38.0047

**Published:** 2024-06-24

**Authors:** Ricardo Barbosa LIMA, Ana Paula Gomes e MOURA, Paulo NELSON-FILHO, Léa Assed Bezerra da SILVA, Marília Pacífico LUCISANO, Raquel Assed Bezerra da SILVA

**Affiliations:** (a) Universidade de São Paulo – USP, School of Dentistry of Ribeirão Preto, Graduate Program in Pediatric Dentistry, Ribeirão Preto, SP, Brazil.; (b) Universidade de São Paulo – USP, School of Dentistry of Ribeirão Preto, Department of Pediatric Clinics, Ribeirão Preto, SP, Brazil.

**Keywords:** Dental Care for Children, Pediatric Dentistry, Ambulatory Care, Unified Health System, Epidemiology

## Abstract

Brazil’s public healthcare system (SUS) offers specialized oral health services to Brazilians, but the productivity of specialists, such as Pediatric Dentists, has not been characterized. Therefore, the objective of this study was to characterize the outpatient dental procedures (ODPs) carried out by Pediatric Dentists within the SUS. An epidemiological study with an ecological, longitudinal, retrospective, and quantitative approach was conducted. The ODPs carried out by Pediatric Dentists within the SUS were characterized based on type of procedure, complexity level, and circumstance (urgent or elective). Data were analyzed using a descriptive and analytical approach, considering a significance level of 5%, as well as the impact of the COVID-19 pandemic (the 2020-2022 years were not included in secondary analyses). In the last 15 years, 29,234,972 ODPs were carried out by Pediatric Dentists within the SUS. Clinical procedures were the majority (55.4%), significantly more frequent than all other types of procedures (all *p* <0.05). Among these, restorative and periodontal procedures were the most common (30.7% and 21.0%, respectively). From 2008 to 2019, excluding COVID-19 pandemic years, the trend over the last 15 years was constant for all types of procedures (all p >0.05). In addition, low complexity ODPs were the majority (90.1%), significantly more frequent than medium (9.7%) and high complexity procedures (0.1%) (both *p* <0.05). At last, most ODPs were not characterized by circumstance in the outpatient production reports (96.9%). Therefore, it was possible to conclude that Pediatric Dentists carried out diverse ODPs within the SUS over the past 15 years, although there was a dominant pattern of type and complexity.

## Introduction

In 2003, one of the objectives of the creation of the National Oral Health Policy (*Brasil Sorridente*) in Brazil was to strengthen specialized/secondary oral health care in Brazil’s public health system (*Sistema Único de Saúde;* SUS), considering the creation and regulation of Dental Specialty Centers (DSCs).^1^ Despite recognizing the need to improve secondary oral health care within the SUS, the offer of dental care in Brazil is focused on Primary Health Care (PHC), with oral health teams included in the Family Health Strategy (FHS) in order to reach users throughout the Brazilian territory, providing resolutivity for most of the population’s oral health demands.^2,3^

Although PHC is the gateway to health care access, and access to oral health services within the SUS has increased significantly over the period after the implementation of *Brasil Sorridente*, the relationship between primary and secondary care still needs to be improved, since unmet demands by oral health teams from the FHS are transferred to secondary care (specialized dental care), such as DSCs.^4,5^ Indeed, the DSCs are the main oral health service associated with secondary level of dental care within the SUS, whose competence must be associated with the management of more complex demands that go beyond the technological density of PHC-linked oral health services, which operate in a referral and counter-referral system. To this end, it was defined that DSCs should provide oral cancer prevention actions (stomatology) and specialized care in periodontics, endodontics, minor oral surgery, and care for people with disabilities.^5,6^

Among the challenges related to DSCs, users’ access to dental specialists/specialties is a relevant problem when considering the resolutivity of secondary care and user satisfaction within the SUS. Although a minimum number of specialties is recommended, the waiting times for appointments, the absence of specialists, the specific needs of the population, and the actions carried out by the DSCs may vary in the Brazilian regions. Thus, disparities in the structures and processes of dental care may arise, drawing attention to the oral health assistance offered at this level of care.^6,7^ It is important to consider the organization of oral health care networks (including its extent and quality) to achieve comprehensive care (integrality) at all levels of care.^3,6^

Furthermore, in addition to the context of health care levels in Brazil’s public healthcare system, there is evidence of the need to evaluate the specialized dental care for children within the SUS,^8^ as well as the inclusion of Pediatric Dentists in the list of mandatory specialties in the DSCs.^9^ This evidence considered the need to promote actions by Pediatric Dentists in secondary care, especially in the DSCs, as a way to address and strengthen children’s oral health and complement PHC. Among other problems, Brazil still faces the high frequency of dental caries in children, especially in socioeconomically vulnerable populations and territories.^8,9^ Although there is a report of specialized dental care for children in DSCs,^10^ as far as it was possible to verify, there is no in-depth characterization of the actions carried out by Pediatric Dentists within the SUS.

This characterization may be useful to understand what actions have been carried out by these specialists within the SUS, whether in the DSCs or in other health services, as secondary level of care (specialized dental care for children). Therefore, the objective of this study was to characterize outpatient dental procedures (ODPs) carried out by Pediatric Dentists within Brazil’s public healthcare system. In addition to providing a descriptive assessment, three alternative hypotheses were evaluated: in the last 15 years, most ODPs procedures carried out by pediatric dentists were clinical procedures (H_1_), most were of medium and high complexity, regardless of the type of procedure (H_2_), and most were elective (H_3_).

## Methodology

### Design

An ecological, longitudinal, retrospective, and quantitative study was conducted.^11^ The approach was delimited to evaluate SUS-related data throughout the Brazilian territory, without distinction of macro-regions. The interval was from 2008 to 2022, considering data availability. To guide scientific reporting, the Portuguese version of the STROBE (Strengthening the Reporting of Observational Studies in Epidemiology) items was adapted and used.^12^ To characterize ODPs carried out by Pediatric Dentists within the SUS, the Outpatient Information System (SIA) was used as a data source, provided by the Department of Informatics (DATASUS; Brazilian Ministry of Health).^13^

### Ethics

All information provided by SIA/SUS was available as public domain with open access, and a population-based analysis was conducted with no contact with humans and no identification or location of SUS professionals or users. Thus, there was no need to submit the study for ethical approval, considering the national resolution number 510 of 2016 from the Brazilian National Health Council - Ministry of Health.^14^

### Variables

The variables were the characteristics of ODPs carried out by Pediatric Dentists within the SUS: type of procedure (health promotion and preventive actions, diagnostic, clinical, surgical, and others), complexity (low, medium, and high), and circumstance (elective, urgent, and others). In addition, based on the annual numbers, the time trend and the incidence ratio of new ODPs carried out by these specialists per year were analyzed. Then, to correct for demographic changes over time in Brazil and establish the ODP’s incidence rate per year similar to another study,^15^ the annual numbers were standardized to 100,000 residents aged zero to fourteen years to capture the most common age group of pediatric patients in the dental setting, considering previous studies on DSC and university-based pediatric dental services.^16-18^ Moreover, in the last 15 years, from 2008 to 2022, data were collected yearly, generating 15 observations for each variable (n = 15). There was no restriction regarding type of oral health service, considering all outpatient productivity attributed to specialists in Pediatric Dentistry within the SUS.

### Data collection

The procedure for retrieving the datasets of interest was based on recommendations on the use of SIA to assess oral health actions within the SUS,^19^ as well as on previous similar approaches.^15,20^ All datasets were gathered in May 2023 from DATASUS by the same researcher. To reach the SIA/SUS, the TabNet tool was used, selecting the options: “health care”, “outpatient productivity”, and “by service location - from 2008”. The geographic scope for datasets was defined as “Brazil by region and state”. Within the SIA/SUS system, data on ODPs carried out by Pediatric Dentists were extracted utilizing the “Professional - Brazilian Classification of Occupations” filter with the code #223236. Regarding productivity status (content), procedures classified as “approved” were considered. The year and characteristics of the procedures were adjusted using compatible filters available: type of procedure, complexity, and circumstance. The estimated annual numbers of Brazilian residents aged zero to fourteen years were collected from the Brazilian Institute of Geography and Statistics (IBGE), also retrieved by the TabNet tool, to normalize the datasets.

### Data analysis

JAMOVI (version 2.3.15, Sydney, Australia) and PAST (version 4.03, Oslo, Norway) statistical packages were used for standard descriptive and analytical statistical approaches. The level of significance was set at 5% (α = 0.05).^21^ The time trend was assessed by the Annual Percent Change (APC), following the steps described in Latorre and Cardoso^22^ and Antunes and Cardoso.^23^ Considering the SARS-CoV-2 outbreak, the time trend was examined with and without the COVID-19 pandemic years (2020, 2021 and 2022; n = 12).^24^The comparison of incidence rates was carried out using the Kruskal-Wallis and Dwass-Steel-Critchlow-Fligner post hoc tests (preliminarily) and Generalized Linear Model (GLM) approach (definitely). Considering an overdispersion, a quasi-Poisson distribution was detected, and robust variance adjustment was used. Then, maximum likelihood estimation was used with a logarithmic link-function (Log-likelihood ratio). The variable with the highest incidence in the preliminary assessment was adopted as the reference level in GLM analysis.^21^

## Results

Table 1 presents the types of ODPs carried out by Pediatric Dentists in the Brazilian Unified Health System from 2008 to 2022 per 100,000 residents between zero and fourteen years old. Altogether, in the last 15 years, 29,234,972 ODPs were carried out. When evaluating time trend, there was a significant decrease in the preventive/health promotion ODPs over time, while the other types of procedures were constant. However, after removing the COVID-19 pandemic years, preventive/health promotion ODPs were also stationary over time (p = 0.738), similar to the other types of procedures. Nonetheless, the lowest annual numbers were observed in the first COVID-19 pandemic year (2020). After preliminary comparison of the annual numbers between types of procedures, statistically significant differences were observed (p < 0.001). Clinical and preventive/health promotion ODPs were the majority and higher than the others (all p <0.05).

**Table 1:** Types of outpatient dental procedures carried out by Pediatric Dentists in the Brazilian Unified Health System from 2008 to 2022 per 100,000 residents between zero and fourteen years old (Brazil, 2023).

Variable	Promotion/ Prevention	Diagnostic	Clinical	Surgical	Others
Median	1,746^A^	36^B^	2,462^A^	123^C^	2^D^
(annual)					
Q1	1,441	32	1,696	96	2
Q3	1,974	44	2,749	152	3
IQR	533	12	1,053	56	1
Minimum	327	22	653	59	1
Year	2020	2020	2020	2020	2010
Maximum	2,309	55	3,825	633	5
Year	2017	2013	2019	2019	2016
Overall	11,531,295	265,276	16,188,023	1,231,805	18,573
*fr*_%_	39.4	0.9	55.4	4.2	0.1
β_1_	-0.031	-0.006	-0.006	-0.013	-0.012
	[-0.002, -0.062]	[-0.019, 0.009]	[-0.033, 0.021]	[-0.051, 0.022]	[-0.042, 0.012]
APC	-6.89	N/A	N/A	N/A	N/A
(%)	[-0.46, -13.3]				
R^2^	0.285	N/A	N/A	N/A	N/A
p-value	0.037*	0.356	0.615	0.442	0.397
Tendency	Decreasing	Stationary	Stationary	Stationary	Stationary

*fr*: approximate relative frequency (%). Q1: first quartile. Q3: third quartile. IQR: interquartile range. A/B/C/D: statistically significant difference. APC: Annual Percent Change. β_1_: angular coefficient. []: 95% confidence interval. R^2^: coefficient of determination. N/A: not applicable. *p-value <0.05.

Table 2 presents the complexity of ODPs per 100,000 residents between zero and fourteen years old. Most dental procedures were characterized as low complexity, followed by medium and high. Then, in the preliminary analysis, low complexity ODPs showed the highest annual numbers compared to medium and high complexity ODPs (p < 0.001). There was a significant increase in the medium and high complexity ODPs over time, while low complexity ODPs were stationary. After removing the COVID-19 pandemic years, there was no significant change from the previous analysis across all ODPs complexities.

**Table 2:** Complexity of outpatient dental procedures carried out by Pediatric Dentists in the Brazilian Unified Health System from 2008 to 2022 per 100,000 residents between zero and fourteen years old (Brazil, 2023).

Variable	Low	Medium	High	Unspecified
Median (annual)	4,006^A^	373^B^	4^C^	3
Q1	3,075	340	2	2
Q3	4,476	493	9	4
IQR	1,401	153	7	2
Minimum	785	235	0	1
Year	2020	2008	2009	2010
Maximum	5,294	642	15	5
Year	2014	2019	2016	2016
Sum	26,342,045	2,832,030	39,425	21,472
*fr*_%_	90.1	9.7	0.1	0.1
β_1_	-0.022	0.019	0.121	N/A
	[-0.052, 0.057]	[0.005, 0.035]	[0.024, 0.252]	
APC	N/A	4.47	32.1	
(%)		[1.16, 8.39]	[5.68, 78.6]	
R^2^	N/A	0.401	0.358	
p-value	0.090	0.013*	0.017*	
Tendency	Stationary	Increasing	Increasing	

*fr*: approximate relative frequency (%). Q1: first quartile. Q3: third quartile. IQR: interquartile range. *p-value <0.05. A/B/C: statistically significant difference. β_1_: angular coefficient. []: 95% confidence interval. R^2^: coefficient of determination. N/A: not applicable.

Table 3 presents the incidence rate comparison of different types and complexities of ODPs. As a definitive analysis, clinical and low complexity ODPs were eligible as a reference for this comparison, considering the preliminary analysis (Table 1 and 2). The incidence rate of clinical ODPs was significantly higher than the other types of procedures (all p <0.001), including health promotion/prevention actions, which was not observed in the preliminary comparison (Table 1). The incidence rate of low complexity ODPs was significantly higher compared to medium and high complexity procedures, confirming the preliminary analysis (Table 2).

**Table 3:** Comparison of incidence rates of different types and complexities of outpatient dental procedures carried out by Pediatric Dentists in the Brazilian Unified Health System from 2008 to 2022 per 100,000 residents between zero and fourteen years old (Brazil, 2023).

Comparison	Distribution	IRR	95% confidence interval		p-value*

			Lower	Upper	
**Reference (type of dental procedure)**		**Clinical**			
Intercept	QPO	145	34.7	240	< 0.001
Promotion/Prevention *versus* Clinical		0.707	0.587	0.851	< 0.001
Diagnostic *versus* Clinical		0.016	0.005	0.037	< 0.001
Surgical *versus* Clinical		0.076	0.047	0.116	< 0.001
Others *versus* Clinical		0.001	0.000	0.011	< 0.001
**Reference (complexity)**		**Low-complexity**			
Intercept	QPO	203	44.0	383	< 0.001
Medium *versus* Low		0.109	0.077	0.150	< 0.001
High *versus* Low		0.002	0.000	0.010	< 0.001

IRR: incidence rate ratio. QPO: quasi-Poisson distribution (overdispersion). *p-value < 0.05.

Table 4 presents the frequencies of ODPs. Pediatric Dentists carried out a considerable number of different types of dental procedures over the last 15 years. However, four groups of ODPs accounted for approximately 54.2% (15,835,900) of the entire outpatient productivity: oral health actions (topical application of fluorides, supervised use of fluoridated mouthwashes, supervised tooth brushing, and dental exams with epidemiological purposes; in group), topical application of fluorides (individual/per session), restorations, and periodontal procedures (dental prophylaxis and sub- or supragingival scaling and polishing). Regarding restorations, 3,255,325 (65.5%) were in primary teeth and 1,714,441 (34.5%) in permanent teeth. In pulpectomies, 161,874 (85%) were in primary teeth and 28,593 (15%) in permanent teeth (treatment or retreatment). At last, 789,936 extractions of primary teeth (73.8%) and 280,248 (26.2%) extractions of permanent teeth were recorded. The other ODPs were not classified according to primary or permanent teeth.

**Table 4:** Frequencies of outpatient dental procedures carried out by Pediatric Dentists in the Brazilian Unified Health System from 2008 to 2022 (Brazil, 2023).

Variable	Complexity	fr	%t	%o
Promotion/Prevention				
Oral health actions (in group)*	L/M	4,482,133	38.9	15.3
Topical application of fluorides (individual/per session)	L	2,988,412	25.9	10.2
Dental plaque coloring	L	1,714,067	14.9	5.9
Cariostatic agent application (per tooth)	L	617,86	5.3	2.1
Sealant agent application (per tooth)	L	573,167	5.0	1.9
Temporary sealing of dental cavity	L	546,576	4.7	1.9
Educational activity/guidance (in group/at the primary or secondary health care)	L	538,133	4.7	1.8
Others		70,947	0.6	0.2
Diagnostic				
Periapical/interproximal radiography	L/M	242,274	91.3	0.8
Panoramic radiography	M	10,67	4.0	< 0.1
Occlusal radiography	M	6,648	2.5	< 0.1
Others		5,684	2.1	< 0.1
Clinical				
Restoration (primary or permanent teeth)	L	4,969,766	30.7	17.0
Periodontal procedures**	L	3,395,589	21.0	11.6
First programmatic dental consultation	L	2,340,438	14.4	8.0
Appointments (at the primary or secondary health care)	L/M	2,198,177	13.6	7.5
Radical endodontic procedures***	L/M	1,353,688	8.4	4.6
Conservative endodontic procedures****	L	742,507	4.6	2.5
Urgency dental care (at the primary or secondary health care)	L/M	623,023	3.8	2.1
Others		564,835	3.5	1.9
Surgical				
Tooth extraction (primary or permanent teeth)	L	1,070,184	86.9	3.7
Frenotomy/Frenectomy	L	20,54	1.7	< 0.1
Abscess incision and drainage	M/L	18,932	1.5	< 0.1
Gingivoplasty/Gingivectomy (by sextant)	M	17,711	1.4	< 0.1
Removal of impacted teeth	M	13,921	1.1	< 0.1
Ulotomy/Ulectomy	L	11,002	0.9	< 0.1
Others		79,505	6.5	0.3
Others				
Removable orthodontic/orthopedic appliance	M	6,842	36.8	< 0.1
Space maintainer	M	3,018	16.2	< 0.1
Fixed orthodontic/orthopedic appliance	M	2,019	10.9	< 0.1
Others		6,694	36.0	< 0.1

*fr*: absolute frequency. %*t*: approximated relative frequency (in each type of outpatient dental procedure). %*o*: approximated relative frequency (overall). *topical application of fluorides, supervised use of fluoridated mouthwashes, supervised toothbrushing and dental exams with epidemiological purposes. **dental prophylaxis and sub or supragingival scaling and polishing. ***Access, medication (with or without root canal preparation) and pulpectomy (treatment or retreatment). ****pulp capping and pulpotomy. L/M/H: low-, medium- and high-complexity outpatient dental procedures.

Most ODPs carried out by Pediatric Dentists (96.9%; 28,338,214) were not characterized according to circumstance in the outpatient production reports. However, of the 896,758 (3.1%) characterized, 880,833 (98.2%) were elective, 15,799 (1.8%) were urgent, and 126 were classified as other circumstances. In addition, more than 600,000 urgency dental care appointments were reported, which differs from the previously mentioned estimate of urgent ODPs. It is also noteworthy that orthodontic/orthopedic procedures in the “others” classification refer to the placement of appliances. Monthly control, adjustment and intervention procedures related to orthodontic appliances amounted to 47,039 (most were classified as high complexity).

## Discussion

This study characterized outpatient dental procedures carried out by Pediatric Dentists in Brazil’s public healthcare system. The alternative hypothesis H_1_ was accepted, since clinical ODPs were more frequent than the other types of procedures. However, H_2_ was rejected, since low complexity ODPs were more frequent. H_3_ could not be properly evaluated because the annual number of ODPs without this information within the SUS was too high. In addition, the COVID-19 pandemic significantly affected the time trend of health promotion/prevention actions carried out by Pediatric Dentists within the SUS.

The first step for understanding the findings is to consider that children’s access to oral health services is influenced, among several factors, by the presence of oral diseases, often dental caries and periodontal diseases.^25,26^ This explains the most frequent ODPs that were conducted in the last 15 years, with more than half of the outpatient productivity being oral hygiene instructions, application of fluoride products, restorations, and basic periodontal procedures, all linked to dental caries and/or periodontal diseases, predominantly clinical, and of low complexity.^27^ Although a dominant pattern of ODPs was observed, Pediatric Dentists carried out a variety of procedures in Brazil’s public health system, including surgical, restorative, endodontic, periodontal, and orthodontic ones.

In addition, when examining these outcomes, it is possible to hypothesize that difficulties in dental care in PHC within the SUS may motivate the referral of children to secondary level (specialized dental care), such as unsuccessfully behavioral management of uncooperative children, a challenge that requires qualified actions from oral health professionals and services.^28^ In parallel, a previous study demonstrated the fragility of PHC in providing dental care to children, especially aged five years or less, since almost 1/5 of the oral health teams did not offer this assistance.^29^ Nonetheless, a literature review pointed out that a significant number of children up to five years old may not have proper access to their first dental appointment within the SUS. In addition, the oral health demands of young children are not adequately resolved in PHC and general practitioners have difficulties in managing the behavior of children in this age group.^8^ Therefore, in addition to the availability of Pediatric Dentists, efforts to improve dental care for children in PHC services should be taken, developing ongoing training for Oral Health Teams at this level of care.^29^

However, an important outcome was the predominance of low complexity procedures. The first hypothesis to justify this finding is the classification assigned by SIA/SUS. The major medium complexity dental procedure was “*appointment in specialized care*” (1,721,490; code #0301010048, information not shown), described as “*clinical appointment of health professionals (except physicians) with a higher level in specialized care*”. It is not possible to delimit the reason for this type of appointment, as well as other ODPs arising from this appointment may have been notified separately. At last, the complexity is delimited by the type of dental procedure, with most being categorized as low complexity, regardless of the clinical issues involved. Moreover, some ODP classifications were changed over time, such as intraoral radiographs (periapical and bitewing), which were of medium complexity until August 2020 and were subsequently categorized as low complexity thereafter (which certainly influenced the annual numbers of medium complexity ODPs, as an example).^13,30^

Therefore, when observing the ODPs carried out by Pediatric Dentists within the SUS in the last 15 years, especially the predominance of low complexity procedures, it is reasonable to question whether the specialized dental care is meeting children’s oral health demands unmet by PHC rather than developing secondary level actions properly. This outcome indicates the need to discuss the interface between primary and secondary levels of dental care in Brazil’s public health system. This relationship is not well understood, considering that SUS actions are mostly directed towards PHC. Moreover, it is necessary to review children’s oral health demands, the organization of health care networks, and the availability of human and material resources, since disparities in access to oral health services are not uncommon.^2,8,31^ Nonetheless, in addition to the ODPs directly related to dental caries, the others are in accordance with the clinical routine in Pediatric Dentistry, which often involves endodontic treatments, preventive and interceptive orthodontic interventions, and surgical procedures, such as extractions, frenectomies, and ulectomies.^27,32^

Furthermore, several limitations of the secondary level of care in Brazil’s public health system should not be disregarded, since it is not uncommon for DSCs to fail to meet productivity goals or to have the minimum mandatory specialties,^33,34^ which may imply the incorporation of complementary specialties, such as Pediatric Dentistry, although the productivity analyzed here was not restricted to this type of oral health service. Nonetheless, Brazil faces an unequal distribution of dentists, especially in terms of the availability of specialists within the SUS compared to the private oral health services (although the SUS is a reference for most Brazilians).^31,35^

When addressing the impact of the COVID-19 pandemic on the time trend of ODPs carried out by Pediatric Dentists within the SUS, it was observed that health promotion/preventive actions were stationary until 2019 and reduced after the SARS-CoV-2 outbreak onset. In addition, the lowest annual numbers were observed in 2020 for all types of procedures. This outcome is in accordance with the prioritization of urgent dental procedures and social distancing recommendations,^36,37^ considering that pediatric dental procedures within the SUS were mostly carried out in groups. Moreover, a reduction in specialized dental care provided by Pediatric Dentists during the first year of COVID-19 pandemic has already been demonstrated,^38^ corroborating this outcome. Removing the 2020-2022 interval did not affect the time trend of ODPs in relation to complexity, which may be understandable when considering that some procedures categorized as medium and high complexity were feasible in the pandemic context, such as urgent care appointments (including its resulting ODPs) and radiographic exams.

As limitations, it is important to consider that specialized care for children within the SUS may also have been carried out by other specialists (e.g. Orthodontists, Endodontists or Special Care Dentists), as well as by Pediatric Dentists with other specialties (allowing the performance of ODPs that are not directly associated with Pediatric Dentistry). In addition, under- or misreporting of ODPs, as well as delays in SIA/SUS database feeding with outpatient production reports might have occurred, generating aggregated data in other time intervals (outliers) and affecting the trend estimation over time. At last, it is important to highlight that there were no restrictions regarding location or type of dental service in which Pediatric Dentists worked, and all health services and productivity within the SUS, including DSCs, were considered. Future investigations may address the relationship between ODPs carried out by Pediatric Dentists and oral health demands of children assisted in Brazil’s public health system, as well as the impact of this productivity on children’s oral health indicators.

## Conclusion

Pediatric Dentists carried out diverse types of ODPs within the SUS in the last 15 years. However, most of them were clinical and of low complexity, and had constant patterns over time. There was not enough information whether the procedure’s circumstance of being elective or urgent. These findings contribute to a better understanding of the landscape of specialized dental care for children within Brazil’s public healthcare system.
